# Barriers to utilisation of oral rehydration solution and zinc in managing diarrhoea among under-5 children in Oyo State, Nigeria

**DOI:** 10.1136/bmjpo-2022-001450

**Published:** 2022-06-23

**Authors:** Adeniyi Francis Fagbamigbe, Joseph Juma, John Kariuki

**Affiliations:** 1 Department of Epidemiology and Medical Statistics, Faculty of Public Health, College of Medicine, University of Ibadan, Ibadan, Nigeria; 2 Department of Epidemiology and Biostatistics, School of Public Health, Mount Kenya University, Nairobi, Kenya

**Keywords:** Epidemiology, Qualitative research

## Abstract

Despite its effectiveness, oral rehydration solution (ORS) and zinc use for managing diarrhoea among under-5 children (U5C) is low in Nigeria. We assessed the barriers to utilisation and sources of ORS/zinc in Oyo State, Nigeria. A cross-sectional mixed-methods design was adopted. Of the 1154 mothers in the quantitative study, only 71 (6.2%) reported recent U5C diarrhoea, of which 41 used ORS/zinc. Eleven of these 41 obtained ORS/zinc from private chemists, and six from government hospitals. Topmost barriers to utilisation of ORS/zinc are unavailability, unaffordability and poor awareness. Stakeholders should intensify efforts to sensitise women, and improve the availability and affordability of ORS and zinc therapy.

Nigeria has the highest burden of diarrhoea in sub-Saharan Africa. Compared with a global average of 70.6, the mortality attributed to diarrhoea in Nigeria among under-5 children (U5C) in 2016 is 327.3/100 000 diarrhoea cases.^1^ WHO and UNICEF recommended that any child with diarrhoea symptoms must be given oral rehydration therapy (ORT) and paediatric zinc sulfate dispersible tablets (zinc) within 24 hours.^2 3^ The oral rehydration solution (ORS) is the most common ORT. ORS utilisation is low in Nigeria.^4^ Literature is replete with factors associated with ORS/zinc use in managing diarrhoea among U5C.^4^ However, there is a paucity of data on barriers to utilisation of ORS/zinc in managing diarrhoea among U5C. We identified the barriers to utilisation of ORS/zinc in managing diarrhoea among U5C in Oyo State, Nigeria, and also assessed the outlets/facilities where these commodities were obtained.

A cross-sectional mixed-methods population-based study was conducted using both quantitative and qualitative methods in Oyo State, Nigeria using a case study approach where health programmers and paediatricians were actively engaged in the study planning and implementation. A total of 1154 mothers/guardians selected using three-stage cluster sampling (wards/communities/households) participated in the quantitative arm while purposively sampled one government senior staff member; two paediatricians had key informant interviews and two focus group discussions (FGDs) among nine mothers/guardians each in the qualitative study.

Only 71 (6.2%) reported recent diarrhoea episodes among their children. A total of 41 (57.7%) of these 71 mothers used ORS/zinc, lower than 34% across Nigeria.^4^ The utilisation varied by respondents’ characteristics. Among FGD participants, main barriers are unavailability, unaffordability, poor awareness and spousal approval. Similar patterns were found in the quantitative arm (table 1). All the key informants stated that ORS/zinc was available free of charge in public hospitals, contrary to unavailability reported by FGD participants. Some mothers/guardians did not know where ORS/zinc can be obtained. Of the 41 with recent U5C diarrhoea who used ORS/zinc in the quantitative study, 11 obtained the commodity from private chemists, and 6 each from government hospitals and government health centres (table 2).

**Table 1:** Reasons for not using ORS/zinc

Reasons	Used recently* (n=41)	Didn’t use recently* (n=30)	All* (n=71)
Unavailability	25	22	47
Unaffordability	36	20	56
Unawareness	0	6	6
Spousal approval	0	16	16
Others	0	2	2
None	0	2	1
Total	41	**30**	**71**

*Multiple responses.

ORS, oral rehydration solution.

**Table 2:** Distribution of places where ORS/zinc was obtained

Place	n
Government hospital	6
Government health centre	6
Public mobile clinic	2
Other public sectors	1
Private hospital, clinic	11
Pharmacy	7
Private chemist/ PMS	5
Itinerant drug seller	1
Others	2
Any	41
None	30
Total	71

ORS, oral rehydration solution; PMS, Patient Medicine Sellers.

The most common stated barriers to the use of ORS/zinc in the management of diarrhoea were availability, affordability and awareness. These are corroborated by existing literature.^3 5 6^ ORS/zinc was obtained at hospitals, chemists, pharmacies and markets. To improve the use of ORS/zinc, sensitisation of mothers/guardians who have poor access to health facilities through radio jingles and programmes and distribution of free ORS/zinc should be intensified.^5 6^ There is a need to design community-level behaviour change components to enhance awareness, elimination and management of diarrhoea. There are needs for hospital management to ensure that ORS/zinc stock-outs in public hospitals are eliminated. Finally, further research is necessary to validate key informants’ claim that the commodities are available free of charge, as it contradicts the mothers/guardians’ stand.

## Supplementary Material

Reviewer comments

Author's manuscript
