# Supplementary material for: Barriers to utilisation of oral rehydration solution and zinc in managing diarrhoea among under-5 children in Oyo State, Nigeria
Source: BMJ Paediatr Open. 2022 Jun 23;6(1):e001450. doi: 10.1136/bmjpo-2022-001450 (PMC9226878; doi:10.1136/bmjpo-2022-001450)
Supplement: Author's manuscript [file bmjpo-2022-001450.draft_revisions.pdf]

BMJ Paediatrics Open

BMJ Paediatrics Open is committed to open peer review. As part of this commitment we make the peer review history of every article we publish publicly available.

When an article is published we post the peer reviewers' comments and the authors' responses online. We also post the versions of the paper that were used during peer review. These are the versions that the peer review comments apply to.

The versions of the paper that follow are the versions that were submitted during the peer review process. They are not the versions of record or the final published versions. They should not be cited or distributed as the published version of this manuscript.

BMJ Paediatrics Open is an open access journal and the full, final, typeset and author-corrected version of record of the manuscript is available on our site with no access controls, subscription charges or pay-per-view fees (http://bmjpaedsopen.bmj.com).

If you have any questions on BMJ Paediatrics Open's open peer review process please email info.bmjpo@bmj.com

BMJ Paediatrics Open

Barriers to utilization and sources of oral rehydration salt and zinc in managing diarrhoea among under-five children in Oyo State, Nigeria: A clarion call

Journal:	*BMJ Paediatrics Open*
Manuscript ID	bmjpo-2022-001450
Article Type:	Original research letter
Date Submitted by the Author:	09-Feb-2022
Complete List of Authors:	Fagbamigbe, Adeniyi; University of Ibadan, ; University of Ibadan<br>Juma, Joseph; Mount Kenya University<br>Kariuki, John; Mount Kenya University
Keywords:	Epidemiology, Qualitative research

SCHOLARONE™
Manuscripts

I, the Submitting Author has the right to grant and does grant on behalf of all authors of the Work (as defined in the below author licence), an exclusive licence and/or a non-exclusive licence for contributions from authors who are: i) UK Crown employees; ii) where BMJ has agreed a CC-BY licence shall apply, and/or iii) in accordance with the terms applicable for US Federal Government officers or employees acting as part of their official duties; on a worldwide, perpetual, irrevocable, royalty-free basis to BMJ Publishing Group Ltd ("BMJ") its licensees and where the relevant Journal is co-owned by BMJ to the co-owners of the Journal, to publish the Work in this journal and any other BMJ products and to exploit all rights, as set out in our licence.

The Submitting Author accepts and understands that any supply made under these terms is made by BMJ to the Submitting Author unless you are acting as an employee on behalf of your employer or a postgraduate student of an affiliated institution which is paying any applicable article publishing charge ("APC") for Open Access articles. Where the Submitting Author wishes to make the Work available on an Open Access basis (and intends to pay the relevant APC), the terms of reuse of such Open Access shall be governed by a Creative Commons licence – details of these licences and which Creative Commons licence will apply to this Work are set out in our licence referred to above.

Other than as permitted in any relevant BMJ Author's Self Archiving Policies, I confirm this Work has not been accepted for publication elsewhere, is not being considered for publication elsewhere and does not duplicate material already published. I confirm all authors consent to publication of this Work and authorise the granting of this licence.

Confidential: For Review Only

Barriers to utilization and sources of oral rehydration salt and zinc in managing diarrhoea among under-five children in Oyo State, Nigeria: A clarion call

*Adeniyi Francis Fagbamigbe[1,2], Joseph Juma[2], John Kariuki[2]

[1]Department of Epidemiology and Medical Statistics, Faculty of Public Health, College of Medicine, University of Ibadan, Ibadan, Nigeria
[2]Department of Epidemiology & Biostatistics, School of Public Health, Mount Kenya University, Nairobi, Kenya
*Correspondence: franstel74@yahoo.com, +2348061348165, ORCID: 0000-0001-9184-8258

Keywords: barriers to utilization, diarrhoea, Nigeria, unavailability, unaffordability, under-five children

Word counts: 598

Reference Counts:6

Confidential: For Review Only

Barriers to utilization and sources of oral rehydration salt and zinc in managing diarrhoea among under-five children in Oyo State, Nigeria: A clarion call

Abstract

Despite its effectiveness, Oral Rehydration Salt (ORS) and Zinc use for managing diarrhoea among under-five children (U5C) is low in Nigeria. We assessed the barriers to utilization and sources of ORS/Zinc in Oyo State, Nigeria. Cross-sectional mixed-method design was adopted. Of the 1154 mothers in the quantitative study, only 71 (6.2%) reported recent U5C diarrhoea, of which 41 used ORS/Zinc. Eleven of these 41 obtained ORS/Zinc from private chemist/PMS, 6 each from government hospital and health centre. Topmost barriers to utilization of ORS/Zinc are unavailability, unaffordability and poor awareness. Stakeholders should intensify efforts to sensitize women, improve availability and affordability.

Keywords: barriers to utilization, diarrhoea, Nigeria, unavailability, unaffordability

Confidential: For Review Only

Nigeria ranks top among the countries with the highest burden of diarrhoea in sub-Saharan Africa. Compared with a global average of 70.6, the mortality attributed to diarrhoea in Nigeria among under-five children (U5C) as of 2016 is 327.3 respectively per 100,000 cases of diarrhoea[1]. Whereas, the World Health Organization (WHO) and UNICEF recommended that a child having the symptoms of diarrhoea must be given Oral Rehydration Therapy (ORT) and Paediatric Zinc Sulphate Dispersible Tablets (Zinc) within 24 hours of such notice[2,3]. The Oral Rehydration Salt (ORS) is the commonest ORT. Literature is replete with factors associated with ORS/zinc use in managing diarrhoea among U5C[4]. However, there is still a paucity of data on barriers to utilization of ORS/zinc in managing diarrhoea among U5C. We aimed to identify the barriers to the utilization of ORS and zinc in managing diarrhoea among U5C in Oyo State, Nigeria and to also assess the outlets/facilities where these commodities were obtained.

A cross-sectional mixed-method population-based study was conducted using both quantitative and qualitative methods in the Oyo State of Nigeria using a case study approach whereby stakeholders were actively engaged. A total of 1,154 mothers/guardians selected using three-stage cluster sampling procedure participated in the quantitative arm while purposively sampled one government senior personnel, two paediatricians had KIIs and two FGDs with 9 mothers/guardians each.

Oly 71(6.2%) reported recent diarrhoea episodes among their children. A total of 41(57.7%) of these 71 mothers used ORS/Zinc. The utilization varied by respondents' characteristics. Among the 24 discussants in the FGDS, the main barrier is unavailability, unaffordability, poor awareness and spousal approval. Similar patterns were found in the quantitative arm (Table 1). The key informants at the ministry of health stated that ORS/Zinc was available free of charge in public hospitals. This was alluded to by key informants at hospitals. Some FGD participants didn't know where ORS/Zinc can be obtained. Of the 41 with recent U5C diarrhoea in the quantitative study, 11 obtained the commodity from private chemist/PMS, 6 each government hospital and government health centre (Table 2).

Table 1: Reasons for not using ORS/Zinc

Reasons	*Used recently (n=41)	*Didn't use recently (n=30)	*All (n=71)
Unavailability	25	22	47
unaffordability	36	20	56
Unawareness	0	6	6
Spousal approval	0	16	16

Confidential: For Review Only

Others	0	2	2
None	0	2	1
Total	41	**30**	**71**

*multiple responses

Table 2: Distribution of places where ORS/Zinc were obtained

Place	n
Government Hospital	6
Government Health Center	6
Public Mobile Clinic	2
Other Public Sector	1
Private Hospital, Clinic	11
Pharmacy	7
Private Chemist/PMS	5
Itinerant Drug Seller	1
Others	2
Any	41
None	30
Total	71

The commonest stated barriers to the use of ORS and or Zinc in the management of diarrhoea were availability, affordability and awareness. These are corroborated by existing literature[3,5,6]. ORS and/or Zinc were obtained at hospitals, chemists, pharmacies and markets. To improve the use of ORS/Zinc, sensitization of women who have poor access to health facilities through radio jingles and programs and distribution of free ORS and Zinc should be intensified[5,6]. There is need to design behaviour change components to enhance awareness, elimination and management of diarrhoea. Finally, there are needs for the management of hospitals to ensure that ORS/Zinc stock-outs in public hospitals are eliminated.

Patient and public involvement

Patients were not directly involved in the design of the study. Rather pediatricians and policymakers at the Oyo State Ministry of health contributed to the data collection instruments and the sampling.

Ethics statements

Ethical approval was obtained from the Oyo State Ministry of Health with approval number AD/ 13/479/1696 with written informed consent obtained from all participants.

Confidential: For Review Only

References

1. GBD 2016 Diarrhoeal Disease Collaborators. Estimates of the global, regional, and national morbidity, mortality, and aetiologies of diarrhoea in 195 countries: a systematic analysis for the Global Burden of Disease Study 2016. *Lancet Infect. Dis.* **18**, 1211–1228 (2018).

2. WHO. *Clinical Management of acute diarrhoea: WHO/UNICEF joint statement*. http://www.who.int/iris/handle/10665/68627 (2004).

3. Black, R. E. Progress in the use of ORS and zinc for the treatment of childhood diarrhea. *J. Glob Heal.* **9**, 10–12 (2019).

4. Akinyemi, I. A., Fagbamigbe, A. F., Omoluabi, E., Agunbiade, O. M. & Adebayo, S. O. Diarrhoea Management Practices and Child Health Outcomes in Nigeria: Sub-National Analysis. *Adv. Integr. Med.* **5**, 15–22 (2018).

5. Wagner, Z., Shah, M. & Sood, N. Barriers to use of oral rehydration salts for child diarrhea in the private sector: Evidence from India. *J. Trop. Pediatr.* **61**, 37–43 (2015).

6. Lam, F. *et al.* An evaluation of a national oral rehydration solution and zinc scale-up program in Kenya between 2011 and 2016. *J. Glob. Health* **9**, (2019).

Footnotes

Funding

No funding received

Conflict of interest

The authors declared no conflict of interest

Author contributions

AFF contributed to conception and study design and conducted the statistical analysis and interpretation of results and the drafting of the manuscripts. AFF, JJ and JK reviewed the statistical analysis and revised the manuscript draft. All authors read and approved the final manuscript.

Acknowledgements

Confidential: For Review Only

I acknowledge the study participants: mothers and guardians of the under-five children, the children, the pediatricians, the staff of Ministry of health of Oyo State Nigeria as well as the staff and students of Mount Kenya University, Nairobi.

Availability of data and materials

The data that support the findings of the current study are available on request to the authors

Confidential: For Review Only

BMJ Paediatrics Open

Barriers to utilization and sources of oral rehydration solution and zinc in managing diarrhoea among under-five children in Oyo State, Nigeria: A clarion call

Journal:	*BMJ Paediatrics Open*
Manuscript ID	bmjpo-2022-001450.R1
Article Type:	Original research letter
Date Submitted by the Author:	31-Mar-2022
Complete List of Authors:	Fagbamigbe, Adeniyi; University of Ibadan, ; University of Ibadan<br>Juma, Joseph; Mount Kenya University<br>Kariuki, John; Mount Kenya University
Keywords:	Epidemiology, Qualitative research

SCHOLARONE™
Manuscripts

I, the Submitting Author has the right to grant and does grant on behalf of all authors of the Work (as defined in the below author licence), an exclusive licence and/or a non-exclusive licence for contributions from authors who are: i) UK Crown employees; ii) where BMJ has agreed a CC-BY licence shall apply, and/or iii) in accordance with the terms applicable for US Federal Government officers or employees acting as part of their official duties; on a worldwide, perpetual, irrevocable, royalty-free basis to BMJ Publishing Group Ltd ("BMJ") its licensees and where the relevant Journal is co-owned by BMJ to the co-owners of the Journal, to publish the Work in this journal and any other BMJ products and to exploit all rights, as set out in our licence.

The Submitting Author accepts and understands that any supply made under these terms is made by BMJ to the Submitting Author unless you are acting as an employee on behalf of your employer or a postgraduate student of an affiliated institution which is paying any applicable article publishing charge ("APC") for Open Access articles. Where the Submitting Author wishes to make the Work available on an Open Access basis (and intends to pay the relevant APC), the terms of reuse of such Open Access shall be governed by a Creative Commons licence – details of these licences and which Creative Commons licence will apply to this Work are set out in our licence referred to above.

Other than as permitted in any relevant BMJ Author's Self Archiving Policies, I confirm this Work has not been accepted for publication elsewhere, is not being considered for publication elsewhere and does not duplicate material already published. I confirm all authors consent to publication of this Work and authorise the granting of this licence.

Confidential: For Review Only

Barriers to utilization and sources of oral rehydration solution and zinc in managing diarrhoea among under-five children in Oyo State, Nigeria: A clarion call

*Adeniyi Francis Fagbamigbe[1,2,3], Joseph Juma[2], John Kariuki[2]

[1]Department of Epidemiology and Medical Statistics, Faculty of Public Health, College of Medicine, University of Ibadan, Ibadan, Nigeria
[2]Department of Epidemiology & Biostatistics, School of Public Health, Mount Kenya University, Nairobi, Kenya
[3]Health Data Science, Populational and Behavioural Science, School of Medicine, University of St Andrews, St Andrews, UK
*Correspondence: franstel74@yahoo.com, +2348061348165, ORCID: 0000-0001-9184-8258

Keywords: barriers to utilization, diarrhoea, Nigeria, unavailability, unaffordability, under-five children

Word counts: 598

Reference Counts:6

Confidential: For Review Only

Barriers to utilization and sources of oral rehydration solution and zinc in managing diarrhoea among under-five children in Oyo State, Nigeria: A clarion call

Abstract

Despite its effectiveness, Oral Rehydration Solution (ORS) and Zinc use for managing diarrhoea among under-five children (U5C) is low in Nigeria. We assessed the barriers to utilization and sources of ORS/Zinc in Oyo State, Nigeria. Cross-sectional mixed-method design was adopted. Of the 1154 mothers in the quantitative study, only 71 (6.2%) reported recent U5C diarrhoea, of which 41 used ORS/Zinc. Eleven of these 41 obtained ORS/Zinc from private chemists, and 6 from government hospitals. Topmost barriers to utilization of ORS/Zinc are unavailability, unaffordability and poor awareness. Stakeholders should intensify efforts to sensitize women, improve the availability and affordability of oral rehydration solution and zinc therapy.

Keywords: barriers to utilization, diarrhoea, Nigeria, unavailability, unaffordability

Confidential: For Review Only

Nigeria has the highest burden of diarrhoea in sub-Saharan Africa. Compared with a global average of 70.6, the mortality attributed to diarrhoea in Nigeria among under-five children (U5C) in 2016 is 327.3/100,000 diarrhoea cases[1]. World Health Organization (WHO) and UNICEF recommended that any child with diarrhoea symptoms must be given Oral Rehydration Therapy (ORT) and Paediatric Zinc Sulphate Dispersible Tablets (Zinc) within 24 hours[2,3]. The Oral Rehydration Solution (ORS) is the commonest ORT. ORS utilization is low in Nigeria[4]. Literature is replete with factors associated with ORS/zinc use in managing diarrhoea among U5C[4]. However, there is paucity of data on barriers to utilization of ORS/zinc in managing diarrhoea among U5C. We identified the barriers to utilization of ORS/zinc in managing diarrhoea among U5C in Oyo State, Nigeria and to also assess the outlets/facilities where these commodities were obtained.

A cross-sectional mixed-method population-based study was conducted using both quantitative and qualitative methods in Oyo State of Nigeria using a case study approach whereby health programmers, and paediatricians were actively engaged in the study planning and implementation. A total of 1,154 mothers/guardians selected using three-stage cluster sampling (wards/communities/households) participated in the quantitative arm while purposively sampled one government senior personnel, two paediatricians had key informant interviews (KIIs) and two focus group discussions (FGDs) among 9 mothers/guardians each in the qualitative study.

Only 71(6.2%) reported recent diarrhoea episodes among their children. A total of 41(57.7%) of these 71 mothers used ORS/Zinc, lower than 34% across Nigeria[4]. The utilization varied by respondents' characteristics. Among FGDs' participants, main barriers are unavailability, unaffordability, poor awareness and spousal approval. Similar patterns were found in the quantitative arm (Table 1). All the key informants stated that ORS/Zinc was available free of charge in public hospitals, contrary to unavailability reported by FGD participants. Some mothers/guardians didn't know where ORS/Zinc can be obtained. Of the 41 with recent U5C diarrhoea who used ORS/zinc in the quantitative study, 11 obtained the commodity from private chemists, 6 each government hospitals and government health centres (Table 2).

Table 1: Reasons for not using ORS/Zinc

Reasons	*Used recently (n=41)	*Didn't use recently (n=30)	*All (n=71)
Unavailability	25	22	47
Unaffordability	36	20	56
Unawareness	0	6	6

Confidential: For Review Only

Spousal approval	0	16	16
Others	0	2	2
None	0	2	1
Total	41	**30**	**71**

*multiple responses

Table 2: Distribution of places where ORS/Zinc were obtained

Place	n
Government Hospital	6
Government Health Center	6
Public Mobile Clinic	2
Other Public Sector	1
Private Hospital, Clinic	11
Pharmacy	7
Private Chemist/PMS	5
Itinerant Drug Seller	1
Others	2
Any	41
None	30
Total	71

The commonest stated barriers to the use of ORS/Zinc in the management of diarrhoea were availability, affordability and awareness. These are corroborated by existing literature[3,5,6]. ORS/Zinc were obtained at hospitals, chemists, pharmacies and markets. To improve the use of ORS/Zinc, sensitization of mothers/guardians who have poor access to health facilities through radio jingles and programs and distribution of free ORS/Zinc should be intensified[5,6]. There is need to design community-level behaviour change components to enhance awareness, elimination and management of diarrhoea. There are needs for hospital management to ensure that ORS/Zinc stock-outs in public hospitals are eliminated. Finally, further research is necessary to validate key informants' claim that the commodities are available free of charge, as it contradicts the mothers/guardians' stand.

Patient and public involvement

Patients were not directly involved in the study design. Rather pediatricians and health programmers contributed to the data collection instruments and sampling.

Ethics statements

Confidential: For Review Only

Ethical approval was obtained from Oyo State Ministry of Health (AD/ 13/479/1696) with written informed consent obtained from all participants.

References

1. GBD 2016 Diarrhoeal Disease Collaborators. Estimates of the global, regional, and national morbidity, mortality, and aetiologies of diarrhoea in 195 countries: a systematic analysis for the Global Burden of Disease Study 2016. *Lancet Infect. Dis.* **18**, 1211–1228 (2018).

2. WHO. *Clinical Management of acute diarrhoea: WHO/UNICEF joint statement*. http://www.who.int/iris/handle/10665/68627 (2004).

3. Black, R. E. Progress in the use of ORS and zinc for the treatment of childhood diarrhea. *J. Glob Heal.* **9**, 10–12 (2019).

4. Akinyemi, I. A., Fagbamigbe, A. F., Omoluabi, E., Agunbiade, O. M. & Adebayo, S. O. Diarrhoea Management Practices and Child Health Outcomes in Nigeria: Sub-National Analysis. *Adv. Integr. Med.* **5**, 15–22 (2018).

5. Wagner, Z., Shah, M. & Sood, N. Barriers to use of oral rehydration salts for child diarrhea in the private sector: Evidence from India. *J. Trop. Pediatr.* **61**, 37–43 (2015).

6. Lam, F. *et al.* An evaluation of a national oral rehydration solution and zinc scale-up program in Kenya between 2011 and 2016. *J. Glob. Health* **9**, (2019).

Footnotes

Funding

No funding received

Conflict of interest

The authors declared no conflict of interest

Author contributions

Confidential: For Review Only

AFF conceived and designed and conducted the statistical analysis and results interpretation. AFF, JJ and JK reviewed the statistical analysis and revised the manuscript . All authors read and approved the final manuscript.

Acknowledgements

I acknowledge the study participants: mothers/guardians, under-five children, pediatricians, staff of Ministry of health of Oyo State Nigeria and staff and students of Mount Kenya University, Nairobi.

Availability of data and materials

The data that support the findings of the current study are available on request to the authors

Confidential: For Review Only

BMJ Paediatrics Open

Barriers to utilization of oral rehydration solution and zinc in managing diarrhoea among under-five children in Oyo State, Nigeria

Journal:	*BMJ Paediatrics Open*
Manuscript ID	bmjpo-2022-001450.R2
Article Type:	Original research letter
Date Submitted by the Author:	28-Apr-2022
Complete List of Authors:	Fagbamigbe, Adeniyi; University of Ibadan, ; University of Ibadan<br>Juma, Joseph; Mount Kenya University<br>Kariuki, John; Mount Kenya University
Keywords:	Epidemiology, Qualitative research

SCHOLARONE™
Manuscripts

I, the Submitting Author has the right to grant and does grant on behalf of all authors of the Work (as defined in the below author licence), an exclusive licence and/or a non-exclusive licence for contributions from authors who are: i) UK Crown employees; ii) where BMJ has agreed a CC-BY licence shall apply, and/or iii) in accordance with the terms applicable for US Federal Government officers or employees acting as part of their official duties; on a worldwide, perpetual, irrevocable, royalty-free basis to BMJ Publishing Group Ltd ("BMJ") its licensees and where the relevant Journal is co-owned by BMJ to the co-owners of the Journal, to publish the Work in this journal and any other BMJ products and to exploit all rights, as set out in our licence.

The Submitting Author accepts and understands that any supply made under these terms is made by BMJ to the Submitting Author unless you are acting as an employee on behalf of your employer or a postgraduate student of an affiliated institution which is paying any applicable article publishing charge ("APC") for Open Access articles. Where the Submitting Author wishes to make the Work available on an Open Access basis (and intends to pay the relevant APC), the terms of reuse of such Open Access shall be governed by a Creative Commons licence – details of these licences and which Creative Commons licence will apply to this Work are set out in our licence referred to above.

Other than as permitted in any relevant BMJ Author's Self Archiving Policies, I confirm this Work has not been accepted for publication elsewhere, is not being considered for publication elsewhere and does not duplicate material already published. I confirm all authors consent to publication of this Work and authorise the granting of this licence.

Confidential: For Review Only

Barriers to utilization of oral rehydration solution and zinc in managing diarrhoea among under-five children in Oyo State, Nigeria

*Adeniyi Francis Fagbamigbe[1,2,3], Joseph Juma[2], John Kariuki[2]

[1]Department of Epidemiology and Medical Statistics, Faculty of Public Health, College of Medicine, University of Ibadan, Ibadan, Nigeria
[2]Department of Epidemiology & Biostatistics, School of Public Health, Mount Kenya University, Nairobi, Kenya
[3]Health Data Science, Populational and Behavioural Science, School of Medicine, University of St Andrews, St Andrews, UK
*Correspondence: franstel74@yahoo.com, +2348061348165, ORCID: 0000-0001-9184-8258

Keywords: barriers to utilization, diarrhoea, Nigeria, unavailability, unaffordability, under-five children

Word counts: 598

Reference Counts:6

Confidential: For Review Only

Barriers to utilization of oral rehydration solution and zinc in managing diarrhoea among under-five children in Oyo State, Nigeria

Abstract

Despite its effectiveness, Oral Rehydration Solution (ORS) and Zinc use for managing diarrhoea among under-five children (U5C) is low in Nigeria. We assessed the barriers to utilization and sources of ORS/Zinc in Oyo State, Nigeria. Cross-sectional mixed-method design was adopted. Of the 1154 mothers in the quantitative study, only 71 (6.2%) reported recent U5C diarrhoea, of which 41 used ORS/Zinc. Eleven of these 41 obtained ORS/Zinc from private chemists, and 6 from government hospitals. Topmost barriers to utilization of ORS/Zinc are unavailability, unaffordability and poor awareness. Stakeholders should intensify efforts to sensitize women, improve the availability and affordability of oral rehydration solution and zinc therapy.

Keywords: barriers to utilization, diarrhoea, Nigeria, unavailability, unaffordability

Confidential: For Review Only

Nigeria has the highest burden of diarrhoea in sub-Saharan Africa. Compared with a global average of 70.6, the mortality attributed to diarrhoea in Nigeria among under-five children (U5C) in 2016 is 327.3/100,000 diarrhoea cases[1]. World Health Organization (WHO) and UNICEF recommended that any child with diarrhoea symptoms must be given Oral Rehydration Therapy (ORT) and Paediatric Zinc Sulphate Dispersible Tablets (Zinc) within 24 hours[2,3]. The Oral Rehydration Solution (ORS) is the commonest ORT. ORS utilization is low in Nigeria[4]. Literature is replete with factors associated with ORS/zinc use in managing diarrhoea among U5C[4]. However, there is paucity of data on barriers to utilization of ORS/zinc in managing diarrhoea among U5C. We identified the barriers to utilization of ORS/zinc in managing diarrhoea among U5C in Oyo State, Nigeria and to also assess the outlets/facilities where these commodities were obtained.

A cross-sectional mixed-method population-based study was conducted using both quantitative and qualitative methods in Oyo State of Nigeria using a case study approach whereby health programmers, and paediatricians were actively engaged in the study planning and implementation. A total of 1,154 mothers/guardians selected using three-stage cluster sampling (wards/communities/households) participated in the quantitative arm while purposively sampled one government senior personnel, two paediatricians had key informant interviews (KIIs) and two focus group discussions (FGDs) among 9 mothers/guardians each in the qualitative study.

Only 71(6.2%) reported recent diarrhoea episodes among their children. A total of 41(57.7%) of these 71 mothers used ORS/Zinc, lower than 34% across Nigeria[4]. The utilization varied by respondents' characteristics. Among FGDs' participants, main barriers are unavailability, unaffordability, poor awareness and spousal approval. Similar patterns were found in the quantitative arm (Table 1). All the key informants stated that ORS/Zinc was available free of charge in public hospitals, contrary to unavailability reported by FGD participants. Some mothers/guardians didn't know where ORS/Zinc can be obtained. Of the 41 with recent U5C diarrhoea who used ORS/zinc in the quantitative study, 11 obtained the commodity from private chemists, 6 each government hospitals and government health centres (Table 2).

Table 1: Reasons for not using ORS/Zinc

Reasons	*Used recently (n=41)	*Didn't use recently (n=30)	*All (n=71)
Unavailability	25	22	47
Unaffordability	36	20	56
Unawareness	0	6	6

Confidential: For Review Only

Spousal approval	0	16	16
Others	0	2	2
None	0	2	1
Total	41	**30**	**71**

*multiple responses

Table 2: Distribution of places where ORS/Zinc were obtained

Place	n
Government Hospital	6
Government Health Center	6
Public Mobile Clinic	2
Other Public Sector	1
Private Hospital, Clinic	11
Pharmacy	7
Private Chemist/PMS	5
Itinerant Drug Seller	1
Others	2
Any	41
None	30
Total	71

The commonest stated barriers to the use of ORS/Zinc in the management of diarrhoea were availability, affordability and awareness. These are corroborated by existing literature[3,5,6]. ORS/Zinc were obtained at hospitals, chemists, pharmacies and markets. To improve the use of ORS/Zinc, sensitization of mothers/guardians who have poor access to health facilities through radio jingles and programs and distribution of free ORS/Zinc should be intensified[5,6]. There is need to design community-level behaviour change components to enhance awareness, elimination and management of diarrhoea. There are needs for hospital management to ensure that ORS/Zinc stock-outs in public hospitals are eliminated. Finally, further research is necessary to validate key informants' claim that the commodities are available free of charge, as it contradicts the mothers/guardians' stand.

Patient and public involvement

Patients were not directly involved in the study design. Rather pediatricians and health programmers contributed to the data collection instruments and sampling.

Ethics statements

Confidential: For Review Only

Ethical approval was obtained from Oyo State Ministry of Health (AD/ 13/479/1696) with written informed consent obtained from all participants.

References

1. GBD 2016 Diarrhoeal Disease Collaborators. Estimates of the global, regional, and national morbidity, mortality, and aetiologies of diarrhoea in 195 countries: a systematic analysis for the Global Burden of Disease Study 2016. *Lancet Infect. Dis.* **18**, 1211–1228 (2018).

2. WHO. *Clinical Management of acute diarrhoea: WHO/UNICEF joint statement*. http://www.who.int/iris/handle/10665/68627 (2004).

3. Black, R. E. Progress in the use of ORS and zinc for the treatment of childhood diarrhea. *J. Glob Heal.* **9**, 10–12 (2019).

4. Akinyemi, I. A., Fagbamigbe, A. F., Omoluabi, E., Agunbiade, O. M. & Adebayo, S. O. Diarrhoea Management Practices and Child Health Outcomes in Nigeria: Sub-National Analysis. *Adv. Integr. Med.* **5**, 15–22 (2018).

5. Wagner, Z., Shah, M. & Sood, N. Barriers to use of oral rehydration salts for child diarrhea in the private sector: Evidence from India. *J. Trop. Pediatr.* **61**, 37–43 (2015).

6. Lam, F. *et al.* An evaluation of a national oral rehydration solution and zinc scale-up program in Kenya between 2011 and 2016. *J. Glob. Health* **9**, (2019).

Footnotes

Funding

No funding received

Conflict of interest

The authors declared no conflict of interest

Author contributions

Confidential: For Review Only

AFF conceived and designed and conducted the statistical analysis and results interpretation. AFF, JJ and JK reviewed the statistical analysis and revised the manuscript . All authors read and approved the final manuscript.

Acknowledgements

I acknowledge the study participants: mothers/guardians, under-five children, pediatricians, staff of Ministry of health of Oyo State Nigeria and staff and students of Mount Kenya University, Nairobi.

Availability of data and materials

The data that support the findings of the current study are available on request to the authors

Confidential: For Review Only
